# Down’s syndrome, neuroinflammation, and Alzheimer neuropathogenesis

**DOI:** 10.1186/1742-2094-10-84

**Published:** 2013-07-16

**Authors:** Donna M Wilcock, W Sue T Griffin

**Affiliations:** 1 Department of Physiology, Sanders-Brown Center on Aging, University of Kentucky, Lexington, KY 40536, USA; 2 Donald W. Reynolds Department of Geriatrics, Donald W. Reynolds Institute on Aging, University of Arkansas for Medical Sciences, 629 Jack Stephens Dr., Little Rock, AR 72205, USA; 3 The Geriatric Research Education Clinical Center, Central Arkansas HealthCare System, Little Rock, AR, USA

## Abstract

Down syndrome (DS) is the result of triplication of chromosome 21 (trisomy 21) and is the prevailing cause of mental retardation. In addition to the mental deficiencies and physical anomalies noted at birth, triplication of chromosome 21 gene products results in the neuropathological and cognitive changes of Alzheimer’s disease (AD). Mapping of the gene that encodes the precursor protein (APP) of the β-amyloid (Aβ) present in the Aβ plaques in both AD and DS to chromosome 21 was strong evidence that this chromosome 21 gene product was a principal neuropathogenic culprit in AD as well as DS. The discovery of neuroinflammatory changes, including dramatic proliferation of activated glia overexpressing a chromosome 2 gene product - the pluripotent immune cytokine interleukin-1 (IL-1) - and a chromosome 21 gene product - S100B - in the brains of fetuses, neonates, and children with DS opened the possibility that early events in Alzheimer pathogenesis were driven by cytokines. The specific chromosome 21 gene products and the complexity of the mechanisms they engender that give rise to the neuroinflammatory responses noted in fetal development of the DS brain and their potential as accelerators of Alzheimer neuropathogenesis in DS are topics of this review, particularly as they relate to development and propagation of neuroinflammation, the consequences of which are recognized clinically and neuropathologically as Alzheimer’s disease.

## Introduction

Down syndrome (DS), trisomy 21, is the most common chromosomal defect, with an incidence in the United States of one per seven-hundred and thirty-three live births [1] with the rate of spontaneous abortions among trisomy 21 fetuses being 7-fold that among non-trisomic fetuses. Not only is trisomy 21 the most frequent cause of mental retardation [2,3], due to the extensive number of chromosome 21 genes [4], there is an extremely high incidence of congenital anomalies such as important cardiac and gastrointestinal malformations in trisomy 21 [5]. In the brain, neuritic amyloid-β (Aβ) plaques - a characteristic neuropathological feature of Alzheimer’s disease (AD) - are a virtually certain finding in adults with DS [6-8] and have been noted in some children with DS [9]. For instance, among 12 children with DS, two (ages 8 and 9 years) had Aβ plaques, and among those between the ages of 35 and 45 years, all had neuritic Aβ plaques and other AD pathologies, such as neurofibrillary tangles and glial activation. Although the prediction of AD neuropathological changes at middle age is reported to be a virtual certainty in those with DS, predictions regarding timing of the onset of dementia are less certain, with estimates of approximately 50 to 70% of individuals with DS developing dementia by age 60 to 70 years [10-15].

Identification and characterization of early events that contribute to and or regulate the expression of chromosome 21 gene products that are triplicated in DS is vital if we are to understand the ways in which neurodegenerative cycles and the mechanisms they employ in promotion of the neuropathophysiological progression of sporadic AD and of AD in DS. Three such early events have been reported in DS fetuses and each is related to the others as they induce, and are induced by each other and by cytokines subsequent to neuroinflammatory changes. In particular, these include overexpression of two chromosome 21 gene products - APP [16] and S100B [17] - and the resultant overexpression of the pluripotent neuroinflammatory cytokine IL-1 [17], which is encoded by chromosome 2 genes *IL-1A and IL-1B*[18,19]. Complex interactions between APP, glial activation, S100B, and IL-1 include upregulation of the expression of IL-1α and β by both APP [20] and S100B [21,22], and induction of both APP and S100B by IL-1β [23]. Such interactions have been shown to be elicited by multiple neural insults, each of which is characterized by gliosis-related neuroinflammation and risk for development of the characteristic neuropathological changes of AD [24].

It is now more than twenty years since the report of prominent glial activation with excessive expression of inflammatory cytokines such as the chromosome 21 gene product S100B and the chromosome 2 gene product IL-1 in DS fetuses, infants, children, and adults in DS, and in AD [17,25]. Such glial activation and cytokine overexpression occurs years before the virtually certain appearance at middle age of the Aβ plaques in DS [14]. Accompanying reports demonstrated that IL-1 induces the synthesis of APP in non-neural and neural cells [21,26]; this IL-1 induction of APP was later verified in neurons in *in vitro* and *in vivo* studies that included evidence of IL-1 induction of S100B [23]. As evidence that glial activation and neuronal expression of APP are potentially self-propagating events, IL-1 and S100B both induce microglial and astrocytic activation with overexpression of themselves, as well as of neuronal APP [23,27]. This idea was supported by reports from Barger [20] and Li *et al*. [28] of neuronal stress-induced excessive expression of neuronal APP and elevated release of sAPP, which, in turn, activates microglia and induces their overexpression of IL-1β [20]. Taken together, these reports suggest that glial activation and resulting cytokine overexpression contribute to APP overexpression and in so doing may favor the appearance of Aβ plaques in children with DS. In DS, then, it may be sufficient to merely invoke gene load-related upregulation of neuronal APP and astrocytic S100B as seminal events that may drive neuronal stress, glial activation, and DS-related neuropathological changes characteristic of AD.

Experimental evidence supports the idea that excessive levels of cytokines such as IL-1 and S100B are key factors in development of the neuropathological changes of AD. For instance in the presence of excess IL-1, whether in brains of experimental animals or in purified rodent neurons in cultures, APP is overexpressed [23,29]. It is logical to assume that *in situ* glial activation with excess IL-1β would favor genesis and progression of Aβ plaque maturation [30], increases in synthesis and phosphorylation of the MAPK-p38 necessary for production of hyperphosphorylated tau and neurofibrillary tangle formation [31]. Such neuroinflammatory consequences are consistent with the idea that any entity, which acts to precipitate changes that lead to genesis of the same entity, may become a self-propagating cycle and thus has the potential to become chronic. If so, neuropathogenesis is, at least in part, the result of self-propagating cycles [32]. By analogy, without regard to the diversity of the source of neuronal stress, for example, traumatic brain injury [33], epilepsy [34-36], aging [37,38], or AIDS [39], the downstream consequence is increased risk for development of the neuropathological changes of AD marked by increased expression of neuronal APP [36,40], activation of glia, and neuroinflammatory cytokine expression [20]. Accordingly, exaggeration of such neuronal-glial interaction due to trisomy 21 [7,24,32] would be expected to culminate in neuropathogenic cycles that favor the progression in DS of the neuropathological changes of AD.

The danger of chronic induction of neuroinflammation with its manifestation of glial activation and cytokine overexpression is related to the capacity of proinflammatory cytokines such as IL-1β to self-propagate as they, themselves, activate microglia and astrocytes and further excess expression of IL-1β. In addition to IL-1β induction of the precursors of the principal neuropathological changes in AD, viz., APP [23] for Aβ plaques, S100B for non-sensical growth of dystrophic neurites in plaques [41], synthesis and activation of MAPK-p38 for hyperphosphorylation of tau [42,43], favors formation of neurofibrillary tangles. In addition to favoring formation of these anomalies, IL-1β induces the synthesis and the activity of acetylcholinesterase, thus favoring the breakdown of acetylcholine [28], an important neurotransmitter in learning and memory [44], which is known to be decreased in AD [45,46]. Similarly devastating, excess IL-1β, as observed in DS and AD, is associated *in vitro* and *in vivo* with decreases in the expression of synaptophysin [31], which is a hallmark of the synaptic loss in AD [47,48]. Such neuropathophysiological changes would be expected to further stress neurons, promote more neuroinflammation, and in this way create a self-propagating cycle [40] of ever increasing neuronal stress, dysfunction, and loss [49].

Microglia, regarded as the principal cells of the brain’s innate immune system, were first identified as a unique glial cell subtype in the second decade of the twentieth century by Pio del Rio Hortega [50] and were later identified immunohistochemically using macrophage cell surface markers [51,52]. In the second to the last decade of the century, microglia were shown to be a primary source of the pluripotent immune cytokine IL-1, and this together with the astrocyte-derived neuritogenic cytokine S100B, were held to be responsible for neuroinflammatory responses in very early stages of the development of DS, and by analogy, perhaps in AD [17]. More recently, astrocytes and neurons as well as oligodendrocytes and vascular pericytes have been recognized as participants in cytokine-related neuroinflammatory processes [53].

In AD, microglia expressing some classic activation markers such as major histocompatibility complex (MHC-II) (associated with antigen presentation), CD68 (a lysosomal protein) and CD36 (a class B scavenger receptor) are highly localized to the area immediately surrounding an amyloid plaque or neurofibrillary tangle [54]. While this led some to hypothesize that microglial activation was contributing to the pathology, others proposed they performed a beneficial function in removing Aβ from the brain [55]. On the one hand, correlation between the overexpression of IL-1α in such plaque-associated microglia with plaque formation and progression from immature deposits to mature *senile* plaques suggests that microglia contribute to formation and progression of Aβ plaques from deposits to mature senile plaques [30,37,38,56]. Similarly, overexpression of IL-1α in activated microglia adjacent to neurons bearing neurofibrillary tangles, as well as the role of IL-1 in promotion of hyperphosphorylated tau [31] suggests a role for activated glia and excess IL-1 in both the production of tangle substrate and in the progression from paired-helical, filamentous threads to non-neuronal, cell-shaped flames of neurofibrillary tangles [57].

### Chromosome 21 genes and neuroinflammation

Triplication of even a small region (21q22.3) on the short arm of chromosome 21, which is referred to as the critical region (DSCR), is sufficient to result in the DS phenotype, prominently including mental retardation, and growth retardation, as well as muscle, joint, and facial features characteristic of DS [58-60]. Recognizing the importance of an extra copy of this precise region of chromosome 21 in the pathology of DS does not lessen the potential importance of triplication of those chromosome 21 genes, which are located outside this region, in neuropathogenesis. The *APP* gene does not map to the critical region of chromosome 21, but more than any other gene on chromosome 21, APP has played a seminal role in our understanding of neuropathological changes in AD and DS, as well as in neurological disorders that give rise to precocious development of AD (for review please see [49]). Steve Barger’s discovery of a role for a secreted fragment of APP, sAPP, in synthesis and release of the pro-neuroinflammatory cytokine IL-1 [20], together with studies demonstrating the importance of IL-1 in promotion of neurodegenerative events [32], underscores the importance of APP, over and above its importance as the precursor of the Aβ in the senile plaques characteristic of AD and DS.

### APP

Although the full significance of triplication of the *APP* gene in Alzheimer pathogenesis in DS is still under investigation, it is clear that there is a dramatic overexpression of the *APP* gene product in fetal DS [61], which is accompanied by a similarly dramatic increase in the levels of IL-1 and S100B [17], two neuroinflammation-promoting cytokines that are known to induce the overexpression of APP *in vitro*[21] and *in vivo*[23].

Glenner’s report of sequence homology between the Aβ peptide derived from brains of those with AD or with DS was the first evidence linking AD to DS, and the *APP* gene to chromosome 21 [62,63]. Although the 50% increase in APP expression that would be predicted as a consequence of gene loading (1.5×) in trisomy, may not be sufficient to result in the characteristic pathophysiology of DS, but, together with its induction of IL-1 and the resultant increase in APP, may be responsible, at least in part, for the role of *APP* gene triplication as a determinant of the age at onset of dementia in DS [64] and in the development of the neuropathological changes of AD in DS [65]. In fact, triplication of the *APP* gene alone is associated with the development of early onset AD [65-67]. Findings such as these, together with the prominence of Aβ plaques in Alzheimer neuropathology, led to development of the Amyloid Hypothesis [68], which has served in many ways to expand our understanding of how APP expression could be related to the development not only the Aβ plaque but to a range of the neuropathological features of AD, including neurofibrillary tangles, glial activation, and overexpression of neuroinflammatory cytokines. However, concentration on Aβ plaques and the Amyloid Hypothesis has to some degree led away from the importance of overexpression of APP, itself, in Alzheimer pathogenesis. For example, neuronal stress imposed by a variety of neurological conditions [32], which are associated with increased risk for development of AD, results in overexpression of APP, release of sAPP, glial activation, and overexpression of IL-1 for genesis of each of the precursors of the neuropathological changes of AD, implying that overexpression of APP starts a cascade of events that gives rise to Aβ deposition rather than Aβ being seminal in Alzheimer pathogenesis. An interesting case that underscores the importance of APP overexpression in Alzheimer pathogenesis is that of a mildly retarded 78-year-old patient with partial trisomy 21 that did not include the *APP* gene sequence [69]. This patient, although of extremely advanced age for a person with DS, especially without the characteristic changes of AD [70], showed no clinical signs of dementia, and magnetic resonance imaging (MRI) 6 months before her death showed only minimal cerebral atrophy and no temporal lobe atrophy.

### BACE2

Mapping of the chromosome 21 *APP* gene, from which is cleaved the Aβ fragment present in plaques in DS and AD [61,71], together with the discovery of mutations in chromosome 21 genes that are linked to familial AD [71] and the virtual certain prediction of precocious development of AD present in those with trisomy 21 [14], lent credence to the importance of chromosome 21 genes in the development of AD [68]. This idea was reinforced by the mapping of the β-amyloid cleavage enzyme 2 (BACE2) to the DS critical region of chromosome 21 [72], which in concert with the γ-secretase complex is essential for cleavage of Aβ 40 and 42 from APP [73,74]. On the other hand, α-secretase, which is not encoded on chromosome 21, plays an important function as it cleaves extracellular APP in the middle of the Aβ portion, preventing release of Aβ [75-77], acting as a neuroprotectant [78], and promoting activation of microglia and induction of synthesis and release of the proinflammatory cytokine IL-1β [20]. If BACE2 induced shedding of IL-1 receptor2 (IL-1R2) [79] acts to avail the brain of decoy receptors for IL-1, the levels of IL-1 may be reduced and therefore neuroinflammation responses in DS and AD dampened.

### S100B

This astrocyte-derived cytokine is encoded by a chromosome 21 gene located in the DSCR [80]. It is markedly elevated during normal development [81] and is elevated throughout life in DS [25,82]. Its properties as a neurite extension factor [83] have been suggested as a contributor to normal growth and maintenance of neurons [84]. In excess, as in DS and AD, S100B is associated with marked nonsensical growth of dystrophic neuronal processes [83,85,86], most notably in the neuritic Aβ plaques diagnostic of AD [25,87,88].

Although S100B is important in development of the central nervous system (CNS) [89] and is protective of neurons from insults and promotes survival following injury [84], elevated blood levels of S100B serve as a marker indicative of stroke severity, survival, and progression from hemorrhage to acute thrombosis [90-92] as well as severity of traumatic brain injury [93]. Even seemingly minor head trauma in children and young adults is associated with elevated blood levels of S100B [94,95]. In addition to increased expression of S100B in activated astrocytes in neurological conditions, systemic diseases such as mild to severe liver disease is characterized by dramatic increases in astrocyte-derived S100B expression, encephalopathy, and cognitive decline [96,97].

#### The role of APP and S100B in promoting neuroinflammation

The dramatic increase in the expression of APP and S100B in DS suggests that (i) some gene product on chromosome 21 is dramatically inducing the APP and S100B synthesis in DS and or (ii) some chromosome 21 gene is indirectly inducing such excessive synthesis, via induction of a gene product on another chromosome, which, in turn, induces excessive expression of APP and S100B. Both of these possibilities have been validated in various cell types, including neuronal cells. For example in the first case, S100B induces the synthesis of neuronal APP mRNA and the production of APP [27]. In the second case, Steve Barger provided the first evidence of a link between neuronal stress, APP expression, and neuroinflammation as he showed that the elevated release of the α-secretase cleaved fragment, sAPPα, which accompanies the stress-induced increases in neuronal expression of APP, activates microglia and induces their expression of IL-1β [20]. Together then it may be concluded that both a chromosome 21 gene product, S100B [80], and a chromosome 2 gene product, IL-1β, contribute to the elevation of APP expression in trisomy 21 to a much greater extent than that expected from gene loading.

Finding relationships between the overexpression APP, S100B, and IL-1β led to a series of experiments to explore mechanisms by which activation of glia and the resultant excess levels of neural IL-1β and S100B influence the neuropathogenesis of AD, as well as the AD of DS [98]. This may be especially the case as such dramatic overexpression of APP as that in DS [61] would be predicted to promote self-propagating cycles of more and more production and release of neuroinflammatory cytokines IL-1 and S100B [17] and a resultant increase in APP. The neuronal strain of such dramatic induction of APP synthesis [99], together with challenges imposed by, for example Aβ plaques [40] and or the presence of neuropathological changes such as neurofibrillary tangle formation have been shown to be accompanied by decreases in cellular expression of the total polyadenylated mRNA necessary for translation of the many proteins involved in cell functions [100].

#### Other lesser-studied chromosome 21 gene products and their importance in neuroinflammatory processes

Each of the chromosome 21 genes cited below encode (Table 1) gene products that also regulate expression of neuroinflammatory processes, and or are regulated by neuroinflammatory cytokines. Although their influence on neuroinflammation and its relation to neuropathogenesis is less studied than APP and S100B, their importance in such pathways should not be underestimated.

**Table 1:** A summary of the inflammation-related genes located on chromosome 21

**Gene**	**Protein**	**Function**	**Reference**
*βAPP*	Amyloid beta precursor protein >5 fold overexpression in DS	Neuronal acute phase protein precursor of fragments Aβ in Alzheimer plaques and sAPP for induction of IL-1β	[16,20,63]
*BACE2*	β-site APP-cleaving enzymes-2	Cleaves APP for less Aβ and increases IL-1R2, a decoy protein for excess IL-1 capture	[79]
*S100B*	S100 calcium binding protein astrocyte-derived cytokine	Upregulates IL-1β and βAPP expression, released in response to TNFα	[22,27,82]
*CXADR*	Coxsackie virus and adenovirus receptor	Activation of JNK and p38-MAPK pathways leading to production of M1 cytokines.	[101]
*ADAMTS1*	ADAM metalloproteinase with thrombospondin type 1 motif, 1	Secreted protease known to be induced by IL-1β	[102]
*ADAMTS5*	ADAM metalloproteinase with thrombospondin type 1 motif, 5	Secreted protease known to be induced by IL-1β and TGFβ.	[103]
*TIAM1*	T-cell lymphoma invasion and metastasis 1	Necessary for cytokine- mediated generation of oxidative species through NADPH oxidase.	[104]
*SOD1*	Superoxide dismutase 1	Scavenges superoxide radicals producing H_2_O_2_ and O_2_.	[105]
*IFNAR2*	Interferon α, β, and ω receptor 2	Activates JAK/STAT mediated anti-inflammatory pathway	[106,107]
*IFNAR1*	Interferon α, β, and ω receptor 1	Activates JAK/STAT mediated anti-inflammatory pathway	[106]
*IFNGR2*	Interferon γ receptor 2	Activates JAK/STAT mediated anti-inflammatory pathway	[107]
*RIPK4*	Receptor-interacting serine-threonine kinase 4	Necessary for signaling through TNFR1	[108]
*CBS*	Cystathione-β-synthase	Catalyzes production of hydrogen sulfide (H_2_S) bimodal regulation of inflammation	[109]
*PRMT2*	Protein arginine methyltransferase 2	Blocks the actions of NFκB in the nucleus	[110]

*Aβ* β-amyloid, *APP* precursor protein for β-amyloid, *IL* interleukin, *MAPK* mitogen-activated protein kinase, *TNF* tumor necrosis factor, *TGF* transforming growth factor, *JAK/STAT* Janus kinase signal transducer and activator of transcription, *TNFR* tumor necrosis factor receptor, *NFκB* Nuclear factor-kappa B.

### CXADR

The chromosome 21 gene that encodes the receptors for coxsackie virus and adenovirus (CXADR) acts as both a viral receptor and as an adhesion molecule associated with tight junctions. It is highly expressed in brain as well as systemic secretory organs such as the pancreas, testis, and small intestine [111]. In the absence of viral infection CXADR expression is elevated in models of myocardial inflammation. In view of triplication of the CXADR gene in trisomy 21 it will be interesting to discover whether there is a pro-inflammatory role for CXADR in the brain similar to that in the heart [112]. This is particularly interesting as CXADR was recently shown to induce stress-activated MAPK pathways in the heart that result in increased production of IFNγ, IL-12, IL-1β, TNFα, and IL-6 [101] as well as changes in tight junction in cardiac vasculature [113]. Increased neural expression of CXADR and its induction of IL-1β and MAPK could contribute to reported MAPK-p38-dependent hyperphosphorylation of tau and tangle formation [31,42].

### ADAMTS1 and ADAMTS5

IL-1β induces synthesis of two other chromosome 21 genes that encode two secreted proteinases of the ADAMTS gene family (ADAMTS1 and ADAMTS5) [102,103,114] that degrade extracellular matrix proteins. This interaction between IL-1β and these proteinases may explain, at least in part, the dramatic overexpression of ADAMTS1 in DS [115]. Moreover, such excessive expression of these proteinases in DS may contribute to degradation of extracellular matrix proteoglycans such as aggrecan and versican in DS [116,117].

### TIAM1

The chromosome 21 gene for T-cell lymphoma invasion and metastasis-1 (TIAM1) is a critical regulatory factor in IL-1β-induced synthesis of NADPH oxidase [104], via induction of guanine nucleotide exchange factor for Rac1 [118] that contributes to the activation of Rac1 for the activation of NADPH oxidase [119] in pancreatic β-cells. By analogy, simultaneous excess expression of TIAM1 and IL-1 in DS brain [17,120] may contribute to the dramatic oxidative stress in DS brain [121].

### SOD1

Superoxide dismutase 1 (SOD1) is a potent endogenous neural-relevant copper and zinc binding enzyme that is encoded on chromosome 21 [105] and acts as a soluble cytoplasmic and mitochondrial interspace protein that converts superoxide radicals to molecular oxygen and hydrogen peroxide [122]. Although the SOD1 gene is most commonly associated with anterolateral sclerosis (ALS) as mutation in SOD1 are linked with genetic susceptibility to ALS [123,124]. However, overexpression of non-mutated SOD1 in the absence of a peroxide-detoxifying enzyme in the same cellular compartment promotes oxidative stress [125], suggesting that the elevation of SOD1 due to trisomy 21, if unmatched by increases in the levels of peroxide-detoxifying enzyme, would be detrimental [126].

### IFNAR1, IFNAR2, and IFNGR2

As the genes encoding the receptors for IFNα1 and 2, and for IFN-γ (IFNAR1, IFNAR2, and IFNGR2) are located on chromosome 21 [106,107] and are therefore all subject to triplication in trisomy 21. IFNAR1 and IFNAR2 respond to IFNα, β, or γ and, upon ligand binding, activate the signaling pathway for induction of proinflammatory cytokines, including IL-1β, TNFα, and IL-6 expression [127]. IFNGR2 uses the same signaling pathway but uniquely responds to IFNγ. In the trisomy 16 mouse model of DS, both the IFNGR2 and IFNAR2 are triplicated. Studies in these mice, which develop significant pathology *in utero* and rarely survive to birth, show that treatment of trisomy 16 fetuses with anti-IFN IgG improves the mouse phenotype, suggesting that the triplication of the IFN receptors significantly contributes to the lethal pathology present in these mice [128]. Furthermore, a partial knockout of *IFNAR2* and *IFNGR2* improves growth and viability of cultured neurons derived from trisomy 16 mouse fetuses [129]. Therefore, the expected hyper-responsiveness to IFN in individuals with DS may contribute to the elevated inflammatory profile in the brain as well as systemically.

### RIPK4

Receptor-interacting serine-threonine kinase 4 (RIPK4) is a protein kinase involved in multiple cell signaling pathways. One of these pathways is the signaling pathway for the activation of NFκB [130,131], which is important in promoting proinflammatory cytokines such as TNFα [132]. In addition, RIPK4 is involved in the signaling cascade of the TNFα receptor TNFR1 [108]. It is important to note that TNFR1 is most heavily implicated in the toxic effects of TNFα, suggesting that overexpression of RIPK4 increases responsiveness of TNFR1 to TNFα, exacerbating the effects of TNFR1.

### CBS

The chromosome 21 gene product cystathionine beta synthase (CBS) [109] is a cytosolic enzyme that catalyzes the desulfhydration of cysteine for production of hydrogen sulfide, which is now recognized as an atypical cellular messenger that is important in both normal [133] and abnormal cellular functions [134]. Hydrogen sulfide is a complicated signaling molecule with an apparent bimodal action on inflammation, in that low levels appear to be anti-inflammatory, while high levels appear to exacerbate neuroinflammatory processes (for review please see [135,136]). Overexpression of CBS in trisomy 21 and its relation to development of AD in DS is, at present, unknown.

### PRMT2

The enzyme, protein arginine methyltransferase 2 (PRMT2), facilitates the methylation of arginine necessary for regulation of the JAK/STAT signaling pathway, which leads to elevation of expression of neuroinflammatory cytokines IFNγ, IFNα, and IL-6 [137] and via inhibition of NFκB may promote [110] apoptosis. Importantly, degradation of proteins containing methylated arginine results in the production of asymmetric dimethylarginine (ADMA) [138], inhibition of nitric oxide synthase activity, and decreased levels of nitric oxide (NO) [139]. If there is an increase in tissue levels of ADMA in DS brain, as there is in the plasma of DS patients with pulmonary hypertension [140], brain levels of NO may be decreased, the JAK-STAT pathway increased, and changes occur toward the inflammatory state of microglia.

### Summary

**Figure 1:**
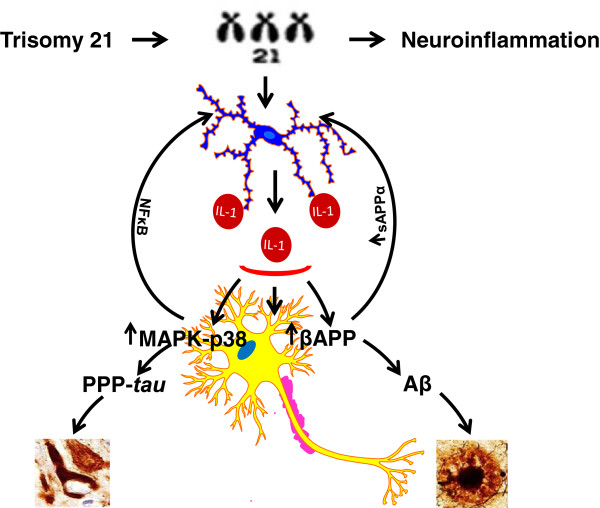
**Schematic highlighting the importance of inflammation-associated genes in the promotion of Alzheimer neuropathogenesis in trisomy 21.** Chromosome 21 genes triplicated in trisomy 21 activate microglia with overexpression and release of proinflammatory cytokines, especially IL-1β, which, in turn, induces further increases in precursor protein for β-amyloid (APP), favoring β-amyloid (Aβ) plaque deposition, and in mitogen-activated protein kinase (MAPK)-p38-dependent phosphorylation and production of phosphorylated tau, favoring neurofibrillary tangle formation, and through nuclear factor κB (NFκB) activity such changes sustain neuroinflammatory responses and consequent neuropathological change.

The principal conclusion one may draw from information presented here regarding the influence of triplication of chromosome 21 genes on neuropathogenesis and development of the clinical and neuropathological manifestations of AD and AD in DS is that many chromosome 21 genes upregulate classical neural immune responses of activated microglia, which are now classified according to systemic immune macrophage profiles as either classical M1 responses that favor overexpression of pro-inflammatory cytokines, or alternative M2 responses that favor overexpression of anti-inflammatory cytokines. As so many chromosome 21 genes are primarily associated with M1, that is, proinflammatory responses, we propose that M1 is the principle glial activation state that will be observed in the DS brain. This is commonsensical as, for example in experimental conditions, triplication of the major IFN receptor genes *IFNAR1, IFNAR2* and *IFNGR2* is associated with enhancement of interferon signaling and TIAM1, SOD1, and PRMT2 are associated with increases in oxidative stresses, and S100B induces expression of APP as well as IL-1α and β. Moreover, in brains of fetuses, neonates, children, and adults with DS, astrocytes are activated and express excess levels of S100B adjacent to activated microglia overexpressing IL-1α and neurons overexpressing APP. These findings, together with triplication of the *βAPP* gene in DS and experimental evidence of neuronal stress-induced increases in βAPP expression and parallel secretion of sAPP for resultant activation of microglia, evidenced by increases in oxidative markers and overexpression and release of IL-1β, suggest that triplication of chromosome 21 genes, perhaps especially *βAPP*, may account for most of the very early neuroinflammatory and oxidative changes in DS. By analogy, the neuronal stress triggered by the many risk factors that favor development of AD, manifested as neuronal overexpression of APP and release of sAPP, may be prime in inducing the dramatic neuroinflammatory responses that then result in a self-amplifying cycle (Figure 1) of increasing neuropathological changes, including neuronal stress, neuroinflammation, and synthesis of the precursors giving rise to formation of the Aβ plaques and neurofibrillary tangles diagnostic of AD.

Relative to other causative factors in individuals at increased risk for development of AD - the small population of individuals with mutations in Aβ-associated genes - trisomy 21 represents the population (one in every seven-hundred and thirty-three live births) with the highest incidence of AD, suggesting that greater attention should be given to investigating DS pathogenesis as it relates to sporadic AD. Such studies could well be directed toward not only discovery of basic molecular mechanisms driven by chromosome 21 gene products but also toward development of rational therapeutic approaches to treatments that may delay onset or slow the progression of AD, thus preserving cognitive functions not only in DS but also in AD itself.

## Abbreviations

Aβ: β-amyloid; AD: Alzheimer's disease; ADMA: Asymmetric dimethylarginine; ALS: Anterolateral sclerosis; APP: Precursor protein of β-amyloid; APP: Precursor protein; BACE2: β-amyloid cleavage enzyme 2; CBS: Cystathionine beta synthase; CNS: Central nervous system; DS: Down’s syndrome; DSCR: Down’s syndrome critical region; IL: Interleukin; IFN: Interferon; IL1-R2: Interleukin 1 receptor 2; IgG: Immunoglobulin; JAK: Janus kinase; JNK: c-Jun N-terminal kinase; MAPK: Mitogen-activated protein kinase; MHC: Major histocompatibility complex; MRI: Magnetic resonance imaging; PRMT2: Protein arginine methyltransferase 2; NAPDH: Nicotinamide adenine dinucleotide phosphate-oxidase; NF-κB: Nuclear factor-kappa B; NO: Nitric oxide; PRMT2: Protein arginine methyltransferase 2; RIPK4: Receptor-interacting serine-threonine kinase 4; SOD1: Superoxide dismutase 1; STAT: Signal transducer and activator of transcription; TIAM1: T-cell lymphoma invasion and metastasis-1 TGF, Transforming growth factor; TNF: Tumor necrosis factor.

## Competing interests

The authors declare that they have no competing interests.

## Authors’ contributions

MW and WSTG, contributed equally to the writing of this review. Both authors read and approved the final manuscript.
